# Delivering oral healthcare for people with cystic fibrosis: A survey of dental practitioners in Ireland

**DOI:** 10.1002/puh2.74

**Published:** 2023-04-09

**Authors:** Fiona O'Leary, Niamh Coffey, Francis M. Burke, Anthony Roberts, Martina Hayes

**Affiliations:** ^1^ Department of Restorative Dentistry Cork University Dental School and Hospital Cork Ireland

**Keywords:** cystic fibrosis, dentists, Ireland, oral healthcare, professional education

## Abstract

**Background:**

Cystic fibrosis (CF) is the most common autosomal recessive condition in Caucasian populations globally. To date, there has been very little research conducted into the oral health of adults with CF possibly due to historic premature mortality. The purpose of this survey was to ascertain knowledge, attitude and practices among dental professionals regarding the oral health of people with cystic fibrosis (PWCF) as part of a larger study being conducted in Ireland.

**Methods:**

A cross‐sectional survey of dental practitioners in Ireland was conducted via an online questionnaire. The survey contained close‐ended questions, and clinical scenarios which allowed for both close‐ended and free text responses in relation to the provision of dental treatment for PWCF. Data was subject to descriptive data analysis using IBM SPSS 29.

**Results:**

The results from the survey indicate a wide variety in the knowledge, attitudes and practices of dental practitioners regarding the oral health of PWCF. There is significant variation in the provision of dental treatment and the conditions under which such treatment is provided.

**Conclusion:**

There is a paucity of information and guidelines specific to this vulnerable population which can make the provision of dental treatment challenging for practitioners. Dental professionals may benefit from continuing professional development and further education targeting patient‐specific populations.

## Graphical abstract

A cross‐sectional study of Irish dental practitioners revealed a paucity of information, guidance and standardized oral healthcare treatment for adults with cystic fibrosis despite the identification of disease‐specific risks to oral health in people with cystic fibrosis.

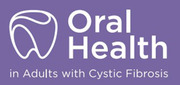

## INTRODUCTION

Cystic fibrosis (CF) is the most common autosomal recessive condition among populations of European descent. It affects approximately 1 in 2500–4000 Caucasian individuals [1]. It is caused by a mutation in both copies of the cystic fibrosis transmembrane conductance regulator (CFTR) gene inherited from each parent. Mutations of the CFTR gene affect proteins that act at the cell surface of all mucous‐producing organs. Therefore, although primarily a disease widely recognized to cause pulmonary illness, it involves multiple organ systems, including the gastrointestinal, reproductive, skeletal and hepatic systems [2]. Advances in the pharmacological treatment and management of the disease have resulted in an increased life expectancy for people with cystic fibrosis (PWCF). Once seen as a disease limited to paediatrics, there are now more adults in Ireland with CF than children with CF [3].

Accompanying this demographical shift are new concerns and challenges for PWCF and healthcare providers. An emerging area of interest is the area of oral health among adults with CF. It has been suggested that PWCFs are at a greater risk of dental caries. This has been attributed to a high‐calorie diet, grazing dietary habits, gastroesophageal reflux disease, increased levels of the cariogenic *Streptococcus mutans* and an increased prevalence of developmental defects of enamel [4].

Historically, the area of oral health in CF has been grossly under‐researched, with studies focusing primarily on children with CF. The literature currently provides conflicting evidence on the levels of oral disease amongst the CF population. Studies reported a lower caries experience in children with CF compared to children without CF [5, 6, 7]. In contrast, recent studies have shown a higher caries experience in PWCF, notably in adults with CF compared to non‐CF adults [8, 9, 10]. It is challenging to ascertain a cause for the recent reports of higher caries levels in PWCF. It may result from an older population, lifestyle and pharmacological changes.

For dental healthcare providers, conflicting evidence makes the provision of appropriate dental treatment challenging. It offers no conclusive information on disease levels, disease‐specific risk factors, pharmacological considerations, preventative treatments and the provision of treatment specific for the CF population. A study evaluating the oral health of adults with CF is currently ongoing in Cork University Dental School & Hospital, involving dentists in the Republic of Ireland. One of the aims of this study is to ascertain knowledge, attitude and practices among dental professionals regarding the oral health of PWCF.

## METHODS

### Study design, population and sampling

A cross‐sectional study assessing “*Oral Health in Adults with Cystic Fibrosis*” through the use of an anonymized online questionnaire distributed electronically using Google Forms was conducted. The sampling method used was convenience sampling. Participants were invited via mailing lists from the Irish Association of Oral Surgeons, the Orthodontic Society of Ireland, the Irish Society of Periodontology and social media channels affiliated with the study.

### Study instrument and variables

Survey: The online questionnaire was designed following extensive Public and Patient Involvement engagement with representatives from the dental profession and patient advocates. Prior to the design of the study questionnaire, the authors met with patient advocate representatives from CF Ireland. These discussions centred on the unique oral needs that PWCF had, and their experiences with dental professionals for example, attendance levels, dentists’ knowledge regarding their specific conditions and medications. Patient advocates also voiced concerns regarding cross infection measures that could be undertaken specific to PWCF. As part of professional involvement, the authors met with local dentists working in community, hospital and private practice to ascertain their current level of knowledge regarding CF, pharmacological management of CF, disease‐specific oral risk factors and specific risk factors that may impact on delivering care to PWCF.

The online questionnaire consisted of 56 questions, 34 questions were multiple choice, 5 questions were related to clinical scenarios posed, and the remaining questions allowed participants to provide personalized answers following on from the multiple choice and clinical scenario questions. The questionnaire has been provided as supplementary material for readers.

### Data analysis

Data was subject to descriptive statistical analysis using IBM SPSS 29. Frequency and percentage frequency of close‐ended questions were calculated. Clinical scenarios contained both close‐ended questions and an additional free text response depending on how respondents answered the initial close‐ended question. Free text responses were subject to text analysis to identify patterns, opinions and knowledge.

### Ethical considerations

Ethical approval (ECM 03/2022 PUB) was granted by the Clinical Research Ethics Committee of the Cork Teaching Hospitals (CREC). Participants gave written consent to partake in the survey. All data collected from this survey was anonymous and was stored securely. IP addresses were not collected at any point, meaning that the data provided could not be traced back to participants. Participants were not obliged to answer all questions.

## RESULTS

A total of 138 dental professionals responded to the survey. Three dentists did not give consent. Therefore, data from 135 dental practitioners was collected and included. Survey respondents included general dental practitioners, oral surgeons, periodontists, orthodontists, community dentists and endodontists as shown in Table 1.

**TABLE 1:** Types and quantities of dental treatment provided for people with cystic fibrosis (PWCF) by respondents (*n* = 32).

Type of treatment provided	Quantities of treatment provided
Examination	27
Preventative	23
Restorative	22
Prosthetic	6
Surgical	8
Orthodontic	4
Endodontic	2
Cosmetic/aesthetic	2

*Note*: Respondents choose multiple options.

### Provision of dental care for people with cystic fibrosis

Overall, 24% (*n* = 32) of respondents were currently providing dental care for PWCF, 13% (*n* = 17) did not know, and 63% (*n* = 85) were not currently providing care for PWCF. Of the 24% currently providing dental care, 18% (*n* = 7) were paediatric patients aged 10 years and less, 21% (*n* = 8) were adolescents aged 10–19 years, and 61% (*n* = 23) were adults.

The type and distribution of dental treatments are shown in Table 1.

Most respondents (60%; *n* = 80) had not previously provided care for PWCF. Of the 40% (*n* = 53) who had provided care to PWCF during their career to date, 13% of practitioners (*n* = 8) had provided care for paediatric patients, 23% (*n* = 14) for adolescents and 64% (*n* = 39) for adults. Similar to dentists currently providing care for PWCF, the distribution of historic care largely consisted of examinations (*n* = 45; 84%), preventative (*n* = 33; 62%) and restorative (*n* = 31; 58%) treatments.

### Attitudes

Most (72%; *n* = 97) dentists reported that they were comfortable treating PWCF. However, 28% (*n* = 38) said that they were not comfortable treating PWCF. The main reasons outlined by respondents for not being comfortable treating PWCF were a lack of knowledge, training and advice in disease pathophysiology, pharmacological therapies and risks to the patient specific to CF. One dentist reported being comfortable now, having had experience treating a patient previously with CF who had received a double lung transplant and was medically stable.

### Education and knowledge

Overall, 89% (*n* = 120) of respondents had not received any specific education or training regarding the provision of dental care for PWCF. Of the respondents (11%; *n* = 15) who had received specific education, 67% (*n* = 10) received this at undergraduate level, whereas 33% (*n* = 5) received it at postgraduate level. Overall, 27% (*n* = 35) of respondents were familiar with the nutritional requirements of PWCF shown in Table 2.

**TABLE 2:** Number of respondents familiar with the nutritional requirements for people with cystic fibrosis (PWCF).

Nutritional requirements of PWCF	Respondents familiar with requirements (*n* = 35)
Increased daily calorie intake	29
Increased frequency of snacking	22
Nutritional supplements	19
Pancreatic enzyme replacement therapy	20

*Note*: Some respondents choose multiple options.

### Special precautions undertaken for PWCF

Regarding additional or special precautions taken to provide dental care for PWCF, 73% (*n* = 96) of dentists did not undertake any specific precautions when treating PWCF. The 27% (*n* = 35) of respondents that did offer additional safeguards to PWCF reported one or a combination of the following: minimizing contact with other patients, shortening appointment times, treating patients in an upright position, allocating PWCF the first or last appointment of the session and ensuring that all members of staff are healthy harbouring no respiratory illness. Overall, 9% (*n* = 11) of dentists were aware that PWCFs often request the first appointment of the clinical session, with 14% (*n* = 18) having practice protocols in place for appointment allocation of appointments for PWCF.

### Medication‐related complications

Respondents were not routinely exposed to the undesirable side effects of the pharmacological management of CF. Only 4% (*n* = 5) of dentists reported patients presenting with tetracycline staining, 78% (*n* = 101) reported PWCF had no tetracycline staining, and 18% (*n* = 24) were unsure if patients had staining. When asked about the prevalence of oral candida in PWCF, 9% (*n* = 12) of dentists reported seeing patients with oral candida, 71% (*n* = 91) reported witnessing no candida, and 20% (*n* = 25) did not know.

Respondents were provided a number of clinical scenarios in the study. They were asked to respond to them.

#### Clinical scenario A

*A patient with Cystic Fibrosis presents requiring caries removal and restoration of a lower molar. The medical history indicates that the patient takes Ivacaftor (gene modulator therapy), CREON (pancreatic enzyme replacement therapy), and inhaled Tobramycin. Are you comfortable administering local anesthetic to this patient?*

Overall, 52% (*n* = 66) of dentists said they would be comfortable administering local anaesthetic; in contrast, 48% (*n* = 62) would not be comfortable. The predominant reason for professionals not being comfortable administering local anaesthetic was insufficient pharmacological information.

#### Clinical scenario B

*A patient with Cystic Fibrosis presents with a buccal sulcus swelling and trismus associated with a non‐vital lower molar. The patient is displaying signs of systemic illness‐ pyrexia and fatigue. The patient takes Tobramycin daily and CREON (pancreatic enzyme replacement therapy). There are no reported allergies. Would you prescribe an antibiotic?*

Overall, 28% (*n* = 35) of respondents would prescribe an antibiotic (amoxicillin 500 mg was the antibiotic of choice for all who responded), 2% (*n* = 2) would not, and 71% (*n* = 90) would seek further advice. This advice would be sought from colleagues (2%; *n* = 2), pharmacists (6%; *n* = 5), general medical practitioners (32%; *n* = 29) and the patient's specialist team (60%; *n* = 54).

#### Clinical scenario C

A 20‐year‐old patient with Cystic Fibrosis requires analgesics following a surgical extraction. The patient is pancreatic insufficient and is taking long‐term inhaled antibiotics for a persistent pulmonary infection. The patient has no allergies. Are you comfortable prescribing analgesics for this patient, or would you seek further advice?

Overall, 23% (*n* = 29) of dentists were comfortable providing analgesics, 5% (*n* = 6) were not satisfied due to drug interactions, and 72% (*n* = 91) would seek further advice before prescribing. When asked who they would liaise with first for further advice on prescribing antibiotics and analgesics for PWCF, 12% (*n* = 11) of respondents indicated the patient's pharmacist, 86% (*n* = 78) would consult the patient's doctor, and 2% (*n* = 2) would consult a dental colleague.

## DISCUSSION

A total of 135 dentists completed the questionnaire. This represents just under 10% of the total number of dentists registered to practice in Ireland. In this study, a considerable percentage of respondents were currently providing (24%; *n* = 32) or had previously provided (40%; *n* = 53) dental care for PWCF. The CF adult population is increasing. The number of registered individuals with CF in Ireland increased from 1130 to 1560 from 2010 to 2019 [11]. The European Respiratory Society and European Cystic Fibrosis Society Task Force on The Provision of Care for Adults with CF in Europe predict that the CF population in Europe will increase by approximately 50% by 2025. This growth will be dominated by an increase in the adult population [12]. An increasing population will retain their teeth for a longer period; this proportion will increase with each age cohort. An ageing dentition can place demands on dental professionals as the burden of oral disease (caries and periodontal disease) increases with age [13].

Despite a low percentage of dental practitioners having received specific training regarding the provision of oral health care for PWCF, most dentists reported that they were comfortable providing care for this cohort. From the results, it appears that PWCF predominantly required preventative care and restorative care, with lesser requiring prosthetic, surgical, endodontic or orthodontic procedures.

Despite 72% (*n* = 97) of dentists reporting that they were comfortable treating PWCF, only 27% (*n* = 35) were familiar with the nutritional requirements of these individuals. Nutritional intake and dietary habits have a significant role in treating CF. The importance of nutritional health on pulmonary health outcomes has been well documented in past and recent literature. A cross‐sectional study conducted from 1971 to 1982 in two large North American CF centres, Toronto and Boston, compared patient characteristics and survival outcomes. Patients treated in the Toronto centre had a greater median survival age (30 years) than those at the Boston centre (21 years). Patients had similar characteristics, but the difference in survival age was attributed to a difference in nutritional status between patients of the two centres. Patients of the Toronto centre were taller and weighed more than their peers at the Boston centre [14].

More recent studies have continued to show the importance of nutrition in treating CF and its effect on disease progression and outcomes. An additional daily calorie intake is necessary for PWCF to compensate for losses due to malabsorption, infection and increased work of breathing. Nutritional guidelines for PWCF recommend that patients eat several small meals and snacks daily [15]. These dietary requirements are typically met by incorporating high fat and sugar contents.

A retrospective review of medical records at Arkansas Children's Hospital CF Care Centre found that children with poorer lung function was associated with lower weight for age percentiles and lower BMI (body mass index) compared to children with higher lung function. Children with poorer lung function had more frequent hospitalizations, sustained poorer nutritional status and had more positive cultures for bacterial colonization [16]. The severity and degree of dental diseases are related to nutritional intake. A diet high in calories or carbohydrate intake can pose significant risks for dental caries.

Dental practitioners are in an advantageous position to highlight the importance of nutrition as part of patient care via personalized patient education, information leaflets and through the analysis of patient diet diaries. The dental community should be mindful of the unique dietary requirements for CF and the potential this poses for caries risk so that they can appropriately advice patients on how to reduce their oral health risk without compromising systemic health.

Regarding special precautions taken for providing dental care for PWCF, 73% (*n* = 96) of respondents did not undertake any specific precautions when treating PWCF. Dental treatment can be offered safely to PWCF in a general dental setting. There are no disease‐specific contraindications for providing dental care to these patients. However, dental practitioners should be aware of complications that may arise from co‐morbidities, notably vitamin K deficiencies, which can prolong post‐operative bleeding, liver disease and bone disease treated with bisphosphonate therapy, and the risk of medication‐related osteonecrosis of the jaw post extraction.

Only 27% (*n* = 35) of respondents did provide additional precautions for PWCF. Precautions such as appointment allocation to minimize patient‐to‐patient contact, for example, the first appointment of the day, are particularly relevant amidst the ongoing COVID‐19 pandemic and in an aerosol‐laden environment and are encouraged by the authors. Furthermore, appointments for non‐sibling PWCF should not be scheduled on the same day to minimize the risk of transmission of pathogens, notably *Pseudomonas aeruginosa*, between patients, which is associated with increased morbidity and mortality [17].

Finally, the respondent's confidence diminished when presented with different clinical scenarios. Just over half of the dentists (52%; *n* = 66) were happy to administer local anaesthetic for restorative treatment to a PWCF taking Ivacaftor, CREON and inhaled Tobramycin, all commonly prescribed medications for CF. The remaining dentists (48%; *n* = 62) would not administer local anaesthetic due to insufficient pharmacological information. Only 28% (*n* = 35) of respondents would administer a broad‐spectrum antibiotic for a PWCF presenting with buccal swelling and trismus. The majority (71%; *n* = 90) would seek further treatment before prescribing.

Similarly, most respondents (72%; *n* = 91) would seek additional information and advice before providing analgesics. The data collected shows shortcomings in the dentist's education specific to PWCF, mainly pharmacological education. More co‐morbidities and medications will accompany an older CF population. Systemic medical conditions can have a negative impact on oral health. In addition, the medications used to treat these conditions may cause oral symptoms. Over the past decade, the CF community has seen dramatic medical management changes, particularly with gene modulator therapies. The medical management of these patients will continue to evolve as treatment progresses, and dental professionals must receive continued education in this developing area. When equipped with knowledge, dental practitioners will be strategically placed to lead advocacy programmes. Examples of such include an online platform promoting oral health that is currently under production for families, carers and patients with CF. This platform will provide free information regarding disease‐specific risks to oral health for PWCF, nutritional advice, oral hygiene advice using various knowledge transfer methods of animations, videos and written posts.

Professional societies, publications and undergraduate training can include education and training aimed at specific populations such as the CF population so that dental professionals are provided with the appropriate tools and knowledge to effectively inform patients on a local level and national level. Patients would also benefit if dental professionals are included as part of the CF multidisciplinary care team. This would allow dentists to incorporate oral health examinations into patient's routine health check‐ups and provide personalized patient focused advice. It would also allow for early identification of vulnerable patients.

The authors have identified that a large proportion of dental practitioners who participated in this study are not comfortable treating PWCF notably when faced with clinical scenarios. There is a lack of information, guidelines and further education available for dental healthcare professionals regarding CF. The development of patient focused oral health policy and guidelines to assist practitioners will improve clinical outcomes and provide an equal platform of care for PWCF.

Several study limitations are important to be addressed. Although the sample size encompasses a broad range of dental specialties, it is small. The questionnaire does not collect specific respondent information, which may help identify why some dental practitioners are more comfortable treating PWCF, such as year of graduation, place of graduation, clinical experience and professional posts. The questionnaire was restricted to dental practitioners who were technologically proficient, as it was not distributed in paper format, so it did not include those who may not have the ability or desire to complete the questionnaire online. Although a valuable tool for qualitative data collection, participant self‐selection may lead to biased data. The respondents who choose to participate may not represent the entire target population. These study limitations could be addressed by undertaking future research to include a larger sample size and distribute the questionnaire in both hardcopy and online versions.

## CONCLUSION

Prevention of oral disease for this population is paramount because oral disease may have detrimental effects in already immunocompromised patients. Professional education and training should be given specifically to CF and other systemic conditions so that dental professionals can provide the appropriate treatment and measure to support the overall systemic health of PWCF and other chronic illnesses.

## AUTHOR CONTRIBUTIONS

*Conceptualization; data curation; formal analysis; investigation; project administration; writing and original draft; writing and review and editing*: Fiona O'Leary. *Data curation; investigation; writing and review and editing*: Niamh Coffey. *Supervision; writing and review and editing*: Francis M. Burke, Anthony Roberts. *Conceptualization; project administration; supervision; writing and review and editing*: Martina Hayes.

## CONFLICT OF INTEREST STATEMENT

The authors declare that they have no known competing financial interests or personal relationships that could have appeared to influence the work reported in this paper.

## ETHICS STATEMENT

This study was conducted in accordance with the UCC research Code of Ethics and inkeeping with the principles of the Declaration of Helsinki.

## Data Availability

The data that supports the findings of this study is available upon email request from the corresponding author Dr Fiona O'Leary.

## References

[1] Fanen P , Wohlhuter‐Haddad A , Hinzpeter A . Genetics of cystic fibrosis: CFTR mutation classifications toward genotype‐based CF therapies. Int J Biochem Cell Biol. 2014;52:94‐102. 10.1016/j.biocel.2014.02.023 24631642

[2] Villanueva G , Marceniuk G , Murphy MS , Walshaw M , Cosulich R . Diagnosis and management of cystic fibrosis: summary of NICE guidance. BMJ. 2017;359:j4574. 10.1136/bmj.j4574 29074599

[3] Keogh RH , Tanner K , Simmonds NJ , Bilton D . The changing demography of the cystic fibrosis population: forecasting future numbers of adults in the UK. Sci Rep. 2020;10(1):10660. 10.1038/s41598-020-67353-3 32606329 PMC7327064

[4] Ferrazzano GF , Sangianantoni G , Cantile T , Amato I , Orlando S , Ingenito A . Dental enamel defects in Italian children with cystic fibrosis: an observational study. Community Dent Health. 2012;29(1):106‐109. 22482260

[5] Primosch RE . Tetracycline discoloration, enamel defects, and dental caries in patients with cystic fibrosis. Oral Surg Oral Med Oral Pathol. 1980;50(4):301‐308. 10.1016/0030-4220(80)90411-9 6935580

[6] Kinirons MJ . The effect of antibiotic therapy on the oral health of cystic fibrosis children. Int J Paediatr Dent. 1992;2(3):139‐143. 10.1111/j.1365-263X.1992.tb00026.x 1304803

[7] Aps JKM , Van Maele GOG , Martens LC . Caries experience and oral cleanliness in cystic fibrosis homozygotes and heterozygotes. Oral Surg Oral Med Oral Pathol Oral Radiol Endod. 2002;93(5):560‐563. 10.1067/moe.2002.121280 12075205

[8] Hildebrandt T , Zawilska A , Trzcionka A , Tanasiewicz M , Mazurek H , Świętochowska E . Estimation of proinflammatory factors in the saliva of adult patients with cystic fibrosis and dental caries. Medicina (Kaunas). 2020;56(11):612. 10.3390/medicina56110612 33202617 PMC7698042

[9] Dabrowska E , Błahuszewska K , Minarowska A , Kaczmarski M , Niedźwiecka‐Andrzejewicz I , Stokowska W . Assessment of dental status and oral hygiene in the study population of cystic fibrosis patients in the Podlasie province. Adv Med Sci. 2006;51(suppl 1):100‐103. 17458069

[10] Pawlaczyk‐Kamieńska T , Borysewicz‐Lewicka M , Śniatała R , Batura‐Gabryel H . Clinical evaluation of the dental hard tissues in an adult population with cystic fibrosis. Pol Arch Intern Med. 2019;129(10):725‐727. 31379359 10.20452/pamw.14918

[11] The Cystic FIbrosis Registry of Ireland . Annual Reports 2020. Cystic Fibrosis Registry Ireland Blog. 2020. https://cfri.ie/annual‐reports/

[12] Schwarz C , Hartl D . Cystic fibrosis in Europe: patients live longer but are we ready? Eur Respir J. 2015;46(1):11‐12. 10.1183/09031936.00026615 26130774

[13] Gavriilidou NN , Belibasakis GN . Root caries: the intersection between periodontal disease and dental caries in the course of ageing. Br Dent J. 2019;227(12):1063‐1067. 10.1038/s41415-019-0973-4 31873267

[14] Corey M , McLaughlin FJ , Williams M , Levison H . A comparison of survival, growth, and pulmonary function in patients with cystic fibrosis in Boston and Toronto. J Clin Epidemiol. 1988;41(6):583‐591. 10.1016/0895-4356(88)90063-7 3260274

[15] Dodge JA . Nutritional requirements in cystic fibrosis: a review. J Pediatr Gastroenterol Nutr. 1988;7(suppl 1):S8‐S11. 10.1097/00005176-198811001-00003 3042941

[16] Quanjer PH , Stanojevic S , Stocks J , et al. Changes in the FEV1/FVC ratio during childhood and adolescence: an intercontinental study. Eur Respir J. 2010;36(6):1391‐1399. 10.1183/09031936.00164109 20351026

[17] Nixon GM , Armstrong DS , Carzino R , et al. Clinical outcome after early *Pseudomonas aeruginosa* infection in cystic fibrosis. J Pediatr. 2001;138(5):699‐704. 10.1067/mpd.2001.112897 11343046

